# Remote Monitoring of Heart Failure in Patients with Implantable Cardioverter-Defibrillators: Current Status and Future Needs

**DOI:** 10.3390/s21113763

**Published:** 2021-05-28

**Authors:** Dominic A. M. J. Theuns, Sumant P. Radhoe, Jasper J. Brugts

**Affiliations:** Department of Cardiology, Erasmus MC, 3015 GD Rotterdam, The Netherlands; s.radhoe@erasmusmc.nl (S.P.R.); j.brugts@erasmusmc.nl (J.J.B.)

**Keywords:** heart failure, remote monitoring, implantable cardioverter-defibrillator, cardiac resynchronization therapy, mortality, hospitalization

## Abstract

The management of heart failure remains challenging despite evidence-based medical and pharmacological advances, especially in the ambulatory setting. There is an urgent need to develop strategies to reduce hospitalizations and readmission rates due to heart failure. Frequent monitoring of high-risk patients is imperative, and with the development of wireless and remote technology, frequent monitoring is now possible via remote monitoring. Nowadays, remote management of patients with cardiac implantable electronic devices is being increasingly adopted and integrated into clinical practice. Several clinical trials studied the impact of remote monitoring on clinical outcomes in patients with implantable cardioverter-defibrillators (ICDs) and cardiac resynchronization defibrillators (CRT-Ds). This point of view will focus on the remote monitoring of ICDs and CRT-Ds in patients with heart failure and discusses whether remote monitoring can be used as a potential instrument for the early identification of patients at risk of worsening heart failure.

## 1. Introduction

Heart failure (HF) is a major and growing public health problem in both Europe and the United States [1]. Despite therapeutic advances, the rates of hospital admissions for HF remain high. HF is the primary diagnosis in >1 million hospitalizations annually [2]. Among beneficiaries of Medicare, a significant proportion of discharged patients with HF are readmitted to the hospital [3]. Patients are usually admitted to a hospital for worsening HF because of signs and symptoms of congestion. Symptoms associated with HF hospitalization are often due to increased filling pressures, which result in pulmonary and systemic venous congestion. Changes in hemodynamics are usually apparent several days to weeks before the onset of symptoms and signs leading to hospital admission. To prevent hospital admissions, patients must be closely monitored to assess changes in physiological parameters related to congestion that may warrant adjustment of HF therapy. Over the last three decades, the management of patients with HF has changed from in-hospital to remote monitoring due to advances in technology [4]. In the late 1980s, telephone-call-based remote assistance was specialized for HF to monitor the status of HF. In the early 2000s, remote monitoring of implantable cardiac devices such as implantable defibrillators (ICDs) was introduced. This technology allows not only continuous monitoring of the integrity of the implanted device, but also the monitoring of some physiological parameters. The monitoring of changes in physiological parameters related to the exacerbation of HF could serve as the basis for early detection of worsening, and may play a key role in HF disease management. The current point of view will highlight the diagnostic capacity of ICD/CRT-D devices in monitoring HF status.

## 2. Remote Monitoring Systems

**Figure 1:**
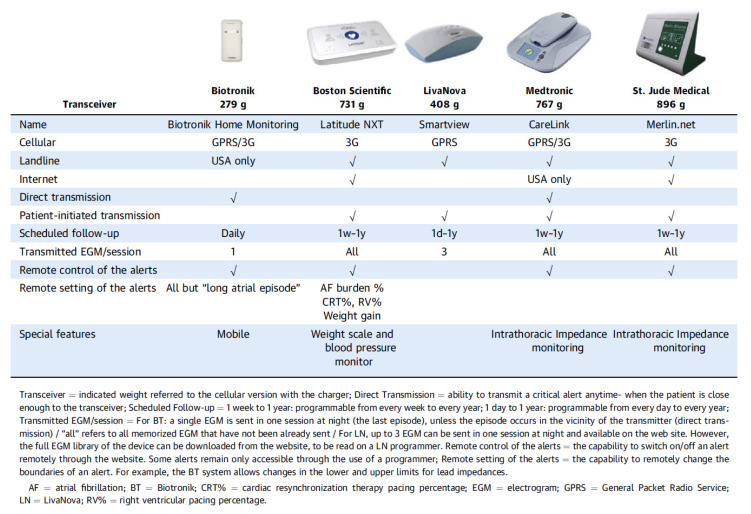
Overview of remote monitoring systems. Reproduced with permission from Ploux et al. (J Am Coll Cardiol EP, 2018) [5].

Contemporary ICD/CRT-D systems are capable of wireless data transmission. Data are transmitted to the manufacturer’s data repository by using either analog or digital landlines or wireless data networks. Remote monitoring of the ICD/CRT-D provides a continuous surveillance of device integrity and shows whether clinical events occurred in addition to remote systematic interrogations. In case of abnormal measurements regarding device integrity or the occurrence of clinical events, alerts may be triggered. These alerts can be programmed either by the programmer or by the website of the respective device manufacturer. The framework of remote patient management is defined by the programmed alerts and the programmed scheduled automatic device interrogations. An overview of the main technical features of the available remote monitoring systems is shown in Figure 1.

## 3. Remote Patient Management

Remote management of patients with an ICD or CRT-D is being increasingly adopted. In the last decade, wireless technology capable of reliable data transmission has extended the reach of applications. A consensus report proposed terms to standardize the descriptions of the different functions of remote patient management [6]. Remote follow-up is defined as a scheduled automatic device interrogation that replaces in-office visits aimed at evaluating device integrity (battery status, lead impedance, sensing, and threshold). Remote monitoring is defined as an automatic transmission of a triggered alert. These alerts can be clinical (e.g., atrial fibrillation or treated ventricular arrhythmias) or technical (e.g., abnormal lead impedance). Patient-initiated interrogation is defined as an unscheduled follow-up initiated manually by the patient in case of real or perceived clinical events.

Data privacy and cybersecurity are important aspects of remote patient management. The General Data Protection Regulation (GDPR) by the European Union (EU) provides a legal framework concerning the collection and processing of personal information. A recent position paper on legal requirements and ethical principles concerning the remote monitoring of cardiac devices recommended a common legal interpretation of the GDPR. Briefly, collecting and retaining data should be limited and specified between the hospital and the manufacturer. A minimum of identifiable data should be collected and processed by manufacturers. Cybersecurity is ensured by all device manufacturers regarding data transfer from the transceiver to the server and hospital.

## 4. Intrathoracic Impedance Monitoring

### 4.1. Single Vector Analysis

**Figure 2:**
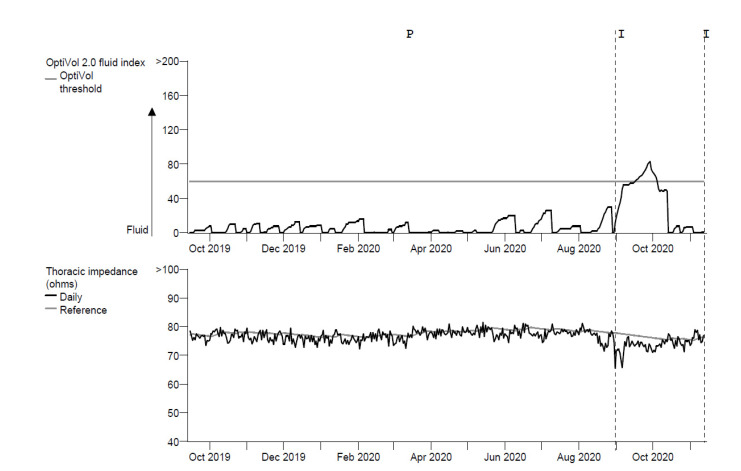
Trend of the OptiVol 2 fluid index and the daily measured intrathoracic impedance (Ohms). The threshold of the fluid index is at the nominal value of 60 (gray horizontal line).

The development of pulmonary congestion can be detected by measuring gradual and progressive changes in intrathoracic impedance. The accumulation of intrathoracic fluid during pulmonary congestion facilitates the conductance of an electrical current, resulting in a corresponding decrease in impedance. By sending a constant current through the tissue using the stimulation electrode pair of the right ventricular high-voltage lead, the resulting voltage and, therefore, the calculated intrathoracic impedance can be acquired from the electrical pathway constructed between the right ventricular coil and the device box. The Medtronic Impedance Diagnostics in Heart Failure (Mid HeFT) feasibility study showed a strong correlation between intrathoracic impedance and pulmonary capillary wedge pressures in hospitalized patients [7]. In the same study, the proposed detection algorithm to detect pulmonary congestion provided an early warning of hospital admissions with 77% sensitivity at a nominal threshold of 60 Ω. These findings were used to develop the OptiVol^TM^ algorithm (Medtronic, Minneapolis, MN, USA), which is employed in Medtronic devices (Figure 2). The utility of this algorithm in detecting HF events in patients with a CRT-D was evaluated in the prospective non-blinded European InSync Sentry observational study [8]. The performance in detecting clinical HF deterioration showed a sensitivity of 60% with a positive predictive value of 60%. The performance of intrathoracic impedance monitoring for the prediction of HF events in chronic HF patients was further evaluated in the prospective, double-blinded Sensitivity of the InSync Sentry OptiVol^TM^ feature for the prediction of Heart Failure (SENSE-HF) study [9]. This study demonstrated a dynamic performance of the algorithm, with a low sensitivity of 21% and a positive predictive value of 5% early after implantation, which both improved over 6 months, producing sensitivity and positive predictive values of 42% and 38%, respectively.

The randomized, controlled Diagnostic Outcome Trial in Heart Failure (DOT-HF) investigated whether monitoring of intrathoracic impedance and other device-based diagnostic information could improve outcomes in patients with HF [10]. Patients were randomized into either an alert arm, in which the physician and patient had access to alerts, or into a control arm without access to alerts. A 79% increase in the HF hospitalization rate was observed in the alert arm when compared to the control arm (*p* = 0.02). In addition, the number of in-office visits was significantly higher in the alert arm compared to the control arm (250 versus 84; *p* < 0.001). In contrast, relatively more signs of HF among control patients were observed during in-office visits. Taken together, the specificity of intrathoracic impedance monitoring alone in detecting HF events was very poor, leading to a high rate of false positive detections and an increased rate of unnecessary in-office visits.

### 4.2. Multiple Vector Analysis

**Figure 3:**
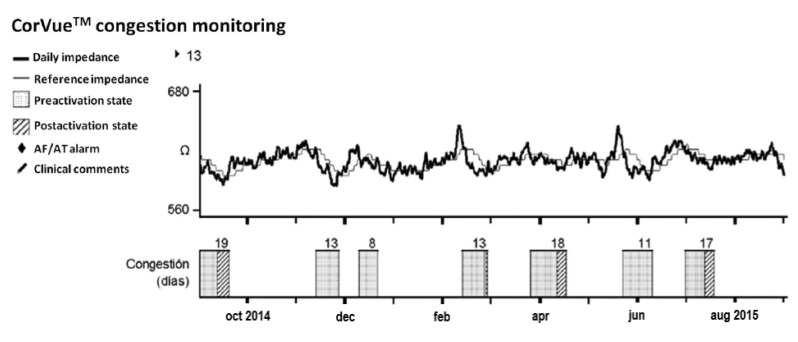
Trend of the CorVue^TM^ congestion monitoring. This example illustrates the potentially misleading information displayed on the device follow-up report. The graphic shows seven episodes of congestion with different days of duration (pre-activation state: checked bars) that generated alarms (post-activation state: oblique lined bars) for four of these episodes without an associated clinical event (false positive). Reproduced with permission from Palfy et al. (Pacing Clin Electrophysiol, 2018) [14].

A possible solution might be to measure changes in impedance by using multiple vectors, which allows the device to capture more of the thoracic tissue than a single right ventricular vector. The CorVue^TM^ system (St Jude Medical, Sylmar, CA, USA) utilizes both right-sided and left-sided electrodes [11]. In a feasibility study that enrolled 75 patients with a CRT-D, a sensitivity of 71.4% and a rate of 0.56 false positive detections per patient-year were found. In comparison, single vector detection had a sensitivity of 57.1% and a rate of 0.74 false positive detections per patient-year [11]. Forleo et al. investigated the performance of the CorVue^TM^ algorithm in 80 patients with heart failure in clinical practice [12]. They observed a sensitivity of 61.5%, with a false positive detection rate of 0.6 per patient-year. Detect Fluid Early from Intrathoracic Impedance Monitoring (DEFEAT-PE) is a prospective, multi-center study of multiple intrathoracic impedance vectors investigating the safety and effectiveness of the CorVue^TM^ algorithm [13]. The algorithm resulted in a low sensitivity of 21.6% and a false positive rate of 0.9 per patient-year. Despite using multiple vectors to detect changes in thoracic impedance, the clinical value of the multi-vector impedance algorithm is limited (Figure 3). Taken together, the diagnostic efficacy of monitoring intrathoracic impedance for early detection of heart failure decompensation is poor, both for single vector (OptiVol^TM^) and multiple vector (CorVue^TM^) algorithms.

## 5. Monitoring Multiple Device Diagnostic Parameters

The identification of several parameters and multiparametric scores able to predict worsening HF may improve the identification of patients at risk of HF events and may facilitate better management strategies for these patients. The Program to Access and Review Trending Information and Evaluate Correlation to Symptoms in Patients with Heart Failure (PARTNERS HF) study was designed to determine the potential utility of multiple device diagnostic parameters in predicting HF events [15]. The device diagnostic parameters included intrathoracic impedance, atrial fibrillation burden, ventricular rate during atrial fibrillation, ventricular tachycardia episodes, patient activity, day and night heart rate, and heart rate variability. An algorithm combining changes in these device diagnostic parameters improved the ability to identify patients at risk of HF events in the next 30 days. A positive HF device diagnostic criterion identified patients who were at risk of experiencing HF events; combined device diagnostics produced a hazard ratio of 5.5 versus only intrathoracic impedance ≥ 60 Ω, with a hazard ratio of 2.7. Based on these findings, an HF risk score was developed that classifies a patient’s risk of HF hospitalization in the next 30 days as high, medium, or low [16]. In the post hoc validation analysis, patients in the high-risk group were 10 times more likely to have an HF hospitalization in the next 30 days compared to those in the low-risk group. Prospective evaluation of the HF risk score has been limited to observational studies with a small sample size [17,18].

Recently, the Multisensor Chronic Evaluation in Ambulatory Heart Failure Patients (MultiSENSE) study evaluated several physiological parameters related to the exacerbation of HF [19]. These parameters included heart sounds, respiration, thoracic impedance, heart rate, and physical activity, which were used to construct a composite index and alert algorithm (HeartLogic^TM^). In the MultiSENSE study, the algorithm effectively detected 70% of worsening HF events with a median early warning of 34 days before the event.

**Figure 4:**
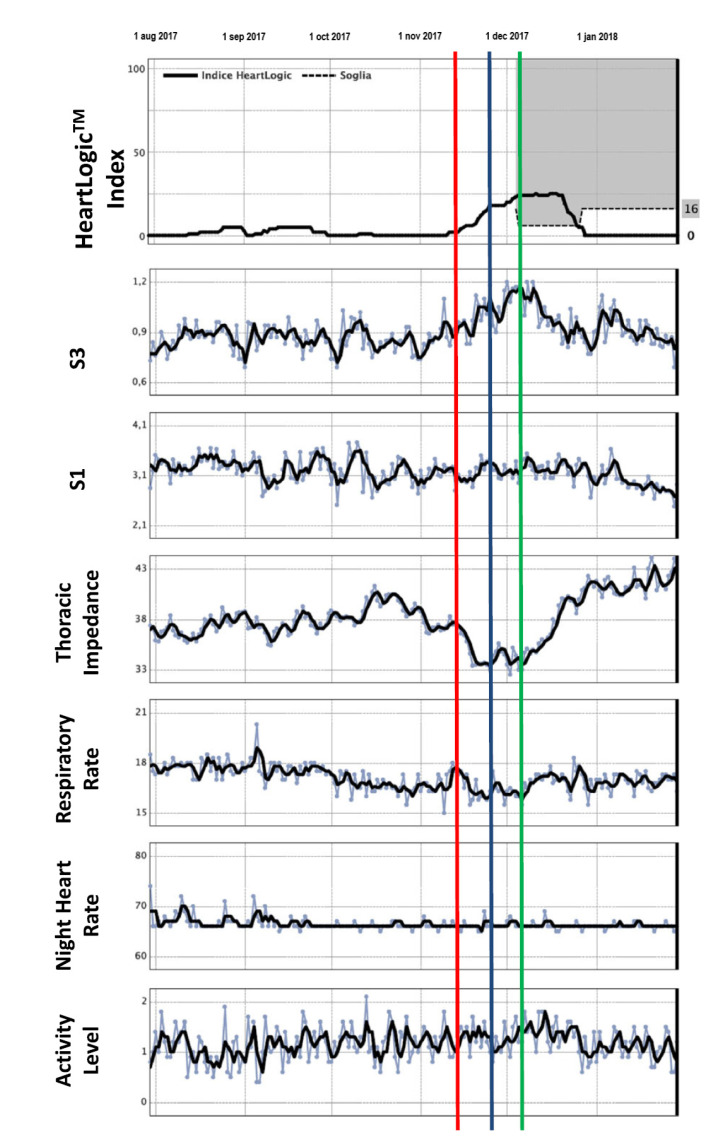
Example of HeartLogic^TM^. At the red line, the patient discontinued diuretic therapy, resulting in weight gain. Diuretic therapy was then restored (green line). The HeartLogic^TM^ index analysis showed crossing of the alarm threshold value, set at a default value of 16 (blue line), with an early warning 10 days in advance compared with clinical evaluations. After therapy restoration, the HeartLogic^TM^ index normalized. Reproduced and modified with permission from Capucci et al. (ESC Heart Fail, 2019) [20].

Both the PARTNERS HF and MultiSENSE studies present promising results, but this multiparametric approach needs further studies to evaluate clinical integration strategies such as remote monitoring and to demonstrate whether this will improve outcomes for HF patients. Recently, the first clinical experience of remote monitoring of HF patients by means of HeartLogic^TM^ was described in a retrospective case series report [20]. The data of 58 patients were analyzed, which encompassed the daily HeartLogic^TM^ index data over a mean follow-up of 5 months. During this follow-up, the default threshold of the index (set at 16) was crossed 24 times in 16 patients, yielding 0.99 alerts/patient-year. An example of the HeartLogic^TM^ index measurement is presented in Figure 4. The median early warning time was 38 days in the case of hospitalizations and 12 days in that of minor events reflecting the clinical deterioration of heart failure, which is similar to the findings in the MultiSENSE study. In this early experience, the HeartLogic^TM^ algorithm demonstrated its ability to detect gradual worsening of heart failure. In order to assess the performance of this algorithm in clinical practice, large studies are needed. Currently, the Multiple Cardiac Sensors for the Management of Heart Failure (MANAGE-HF) trial is recruiting patients to evaluate the performance of HeartLogic-alert-based management in improving mortality and morbidity from HF when used in more routine care (NCT03237858).

## 6. Randomized Clinical Trials and Remote Monitoring

Several randomized clinical trials (RCTs) were conducted to evaluate the overall impact of remote monitoring on clinical outcomes in patients with an ICD or CRT-D [21,22,23,24,25,26,27,28,29] (Table 1). The total number of patients enrolled in the nine RCTs was 8326. The mean age of the patients ranged from 62 to 70 years, with the proportion of male patients ranging from 71% to 88%. The mean left ventricular ejection fraction ranged from 25% to 35%, and the mean proportion of patients with ischemic cardiomyopathy ranged from 44% to 70%. The mean or median follow-up of the RCTs ranged from 12 to 34 months.

The clinical parameters measured by the implanted ICD/CRT-D in the RCTs are presented in Table 2. The majority of RCTs also performed a telemedicine-based disease management strategy—for example, telephone interviews to evaluate the clinical status of the patients by assessing symptoms, dyspnea, weight gain, edema, fatigue, and activity status.

**Figure 5:**
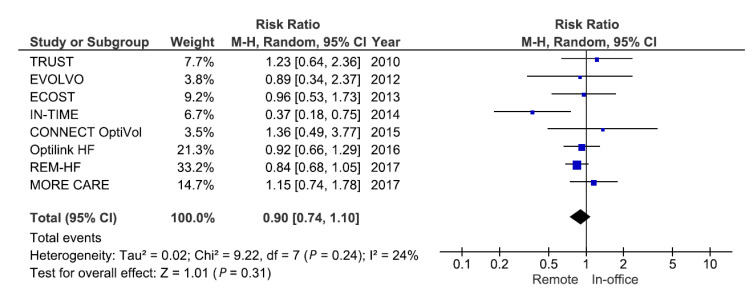
Pooled relative risk for randomized clinical trials examining the effect of remote monitoring on all-cause mortality among patients with an implantable cardioverter-defibrillator or cardiac resynchronization defibrillator. Abbreviations: 95% CI = 95% confidence intervals; M-H = Mantel–Haenszel test.

A total of eight RCTs enrolling 6329 patients reported on all-cause mortality. The pooled risk ratio (RR) for all-cause mortality with remote monitoring was not statistically significant from in-office visits (RR 0.90; 95% CI: 0.74 to 1.10; *p* = 0.31) (Figure 5). Only the Influence of Home Monitoring on mortality and morbidity in heart failure patients with impaired left ventricular function (IN-TIME) study observed a significant reduction in all-cause mortality with remote monitoring (RR: 0.35; 95% CI: 0.17 to 0.73; *p* = 0.005) [25]. When excluding IN-TIME, the RR for all-cause mortality changed marginally (RR: 0.93; 95% CI: 0.80 to 1.08; *p* = 0.35), but eliminated between-study heterogeneity (I^2^ = 0%). A meta-analysis by Parthiban et al. examined the effect of competing remote monitoring technologies on all-cause mortality [30]. When pooling the results of three trials using remote monitoring technology from Biotronik SE & Co. (Berlin, Germany), which uses daily transmission, a reduction in all-cause mortality with remote monitoring was observed (RR: 0.65; 95% CI: 0.45 to 0.94; *p* = 0.02). This result was confirmed by a pooled analysis using patient-level data of the same three RCTs (TRUST, ECOST, and IN-TIME) [31]. At 1-year follow-up, the absolute risk of all-cause mortality was reduced by 1.9% (95% CI: 0.1–3.8%; *p* = 0.037).

**Figure 6:**
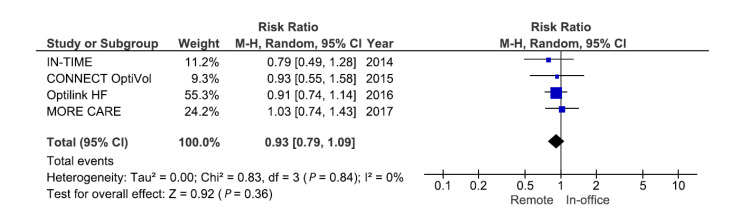
Pooled relative risk for randomized clinical trials examining the effect of remote monitoring on heart-failure-related hospitalization.

Data on HF hospitalization were reported in four RCTs enrolling 2707 patients. The pooled data of these RCTs showed no significant reduction in the relative risk of hospitalization due to HF (RR: 0.93; 95% CI: 0.79 to 1.09; I^2^ = 0%; *p* = 0.36) (Figure 6). CONNECT OptiVol, OptiLink HF, and MORE CARE applied alert-based monitoring based on intrathoracic impedance to monitor HF, while IN-TIME used daily transmission of other parameters (Table 2). When excluding IN-TIME, the relative risk for HF hospitalization changed marginally (RR: 0.95; 95% CI: 0.80 to 1.12; I^2^ = 0%; *p* = 0.53). Alert-based monitoring of intrathoracic impedance or other parameters is not sufficiently sensitive to detect HF deterioration in order to prevent hospitalization.

The Remote Management of HF using implantable electronic devices (REM-HF) trial is the largest study with the longest follow-up on remote monitoring of HF to date [28]. In this trial, no alert-based strategy was used. Instead, changes in trends over time in the monitored parameters were reviewed weekly. A total of 1650 patients were randomly assigned to remote monitoring or usual care, and the median follow-up was 2.8 years. The investigators found no reduction in the risk of all-cause mortality or hospitalization for cardiovascular reasons with management guided by weekly active remote monitoring as compared to usual care (HR: 1.01; 95% CI: 0.87 to 1.18; *p* = 0.87). The monitoring resynchronization devices and cardiac patients (MORE-CARE) study enrolled 865 patients [29]. At 2-year follow-up, no reduction in all-cause mortality or hospitalization for cardiovascular or device-related reasons in the remote arm was observed (HR: 1.02; 95% CI: 0.80 to 1.30; *p* = 0.89). In contrast to REM-HF and MORE-CARE, only the IN-TIME trial provided a reduction in all-cause mortality and HF hospitalization and a change in NYHA score in the remote arm. This reduction was mainly driven by a reduction in mortality, primarily in patients with a history of AF.

## 7. Remote Monitoring, Atrial Fibrillation, and Heart Failure Hospitalization

Atrial fibrillation can be accurately quantified by remote monitoring when an atrial lead is implanted. With this in mind, AF has not only been an important cause of strokes and inappropriate ICD shocks, but has also been linked to increased risk of HF hospitalization [32,33]. In patients treated with CRT, AF may reduce biventricular pacing, which limits the efficacy of the CRT. Therefore, early detection of AF by remote monitoring affords optimization of rate or rhythm control therapies that may prevent AF-related HF decompensation. As mentioned in the previous section, the IN-TIME trial found that remote monitoring primarily improved outcomes in HF patients with a history of AF, which was mainly driven by a reduction in mortality. A recent post hoc analysis of the REM-HF trial evaluated whether a similar reduction in mortality would be present among patients with AF as compared to those in sinus rhythm [34]. In addition, the risk of hospitalization was evaluated to determine whether this was reduced considering recurrent hospitalizations after the first event. The main finding of this post hoc analysis was that the use of remote monitoring to guide HF management for patients with AF was associated with poorer outcomes. The investigators found an increased risk of mortality and more unplanned cardiovascular hospitalizations, mainly due to worsening HF in patients with permanent AF.

The discrepancy between the outcomes of the IN-TIME and REM-HF trials is interesting. Several aspects may explain this discrepancy. First, considering baseline clinical characteristics, patients enrolled in the IN-TIME trial had more advanced HF compared to those in the REM-HF trial and lower mean LVEF (26% versus 30%), and more patients had NYHA functional class III (57% versus 30%). Second, patients with permanent AF were excluded in IN-TIME, while this was not an exclusion criterion in REM-HF. The higher proportion of patients with permanent AF in REM-HF may have mitigated the beneficial effect of remote monitoring. Patients with paroxysmal AF could derive more benefits from remote monitoring by improving rate control or restoring sinus rhythm.

Thus, all aforementioned RCTs have led observers to question the usefulness of remote monitoring in the HF setting. However, these trials were heterogeneous in methodological quality, sample size, severity of HF, centralized monitoring of data, frequency of data transmission, and intervention. Remote monitoring of device data is feasible but the impact is highly dependent on the process of decision-making on remote transmitted data.

## 8. Conclusions

Remote monitoring has a Class I recommendation for the follow-up of patients with an ICD or CRT-D regarding device function and arrhythmia management [35]. However, remote management of HF using thoracic impedance alone or combined with other parameters is still uncertain and has received a Class II-b recommendation [35]. In order to translate the potential advantages of remote monitoring into improved outcomes in HF patients, several aspects must be considered, starting with the parameter or set of parameters that has value in identifying patients at risk of HF events. The monitoring of multiple device parameters combined with an algorithm needs further investigation in large clinical trials. Second, the frequency of transmission of monitored parameters should be decided—daily, weekly, or monthly. The third decision relates to the use of alert-based monitoring or a review of trends in the monitored parameters. When comparing daily versus weekly and alert-based monitoring versus review of trends, both showed no reduction in HF hospitalizations using remote monitoring compared to usual care in HF patients, as shown in the IN-TIME and REM-HF trials. Another factor is the presence of telemedicine using a structured telephone interview to assess the clinical symptoms of the patients and, subsequently, the presence of a treatment plan. Future advances in technology, such as the development of new sensors in devices or wireless connection with hemodynamic sensors, e.g., a pulmonary artery pressure sensor (CardioMEMS), may further improve the management of HF patients [36].

## Figures and Tables

**Table 1:** Baseline characteristics of patients included in randomized clinical trials.

Study	FU (Months)	Sample Size (n)	RM (n)	IO (n)	Age (yrs)	Male (%)	LVEF (%)	ICM (%)	NYHA II (%)	NYHA III-IV (%)
TRUST [21]	12	1,339	908	431	64	73	29	67	57	30
CONNECT [22]	15	1,997	1014	983	65	71	29	62	40	50
EVOLVO [23]	16	200	99	101	67	79	31	46	70	19
ECOST [24]	24	433	221	212	62	88	35	65	62	9
IN-TIME [25]	12	664	333	331	65	82	26	70	43	57
CONNECT OptiVol [26]	15	176	87	89	66	77	32	53	46	43
OptiLink HF [27]	18	1,002	505	497	66	80	27	54	19	81
REM-HF [28]	34	1,650	824	826	70	86	30	68	70	30
MORE-CARE [29]	24	865	437	428	66	76	27	44	38	60

FU = follow-up; ICM = ischemic cardiomyopathy; IO = in-office; LVEF = left ventricular ejection fraction; NYHA = New York Heart Association; RM = remote monitoring.

**Table 2:** Overview of programmed clinical parameters in the randomized clinical trials.

Study	Parameters	Telemedicine-Based Disease Management
TRUST [21]	VT, VF, SVT, ineffective 30-J shock, mode switch duration >10% in 24 h	No
CONNECT [22]	AT/AF burden, ventricular rate during AT/AF, number of shocks delivered, all therapies exhausted in a zone	No
EVOLVO [23]	Thoracic impedance (OptiVol), AT/AF burden, number of shocks delivered	Yes
ECOST [24]	VT, VF, SVT, ineffective 30-J shock, >75% (18 h) spent in mode switch	No
IN-TIME [25]	VT, VF, SVT, % biventricular pacing, PVC/h, patient activity	Yes
CONNECT OptiVol [26]	Thoracic impedance (OptiVol)	Yes
OptiLink HF [27]	Thoracic impedance (OptiVol)	Yes
REM-HF [28]	Thoracic impedance, % biventricular pacing, AT/AF burden, ventricular arrhythmias, activity level, heart rate variability	Yes
MORE-CARE [29]	Thoracic impedance (OptiVol), AT/AF burden	Yes

AF = atrial fibrillation; AT = atrial tachycardia; PVC = premature ventricular complex; SVT = supraventricular tachycardia; VF = ventricular fibrillation; VT = ventricular tachycardia.

## Data Availability

Not applicable.
